# A new rapid diagnostic test for detection of anti-*Schistosoma mansoni* and anti-*Schistosoma haematobium* antibodies

**DOI:** 10.1186/1756-3305-6-29

**Published:** 2013-01-29

**Authors:** Jean T Coulibaly, Eliézer K N’Goran, Jürg Utzinger, Michael J Doenhoff, Emily M Dawson

**Affiliations:** 1 Department of Epidemiology and Public Health, Swiss Tropical and Public Health Institute, P.O. Box, CH-4002, Basel, Switzerland; 2 University of Basel, P.O. Box, CH-4003, Basel, Switzerland; 3 Unité de Formation et de Recherche Biosciences, Université Félix Houphouët-Boigny, 22 BP 770, Abidjan 22, Côte d’Ivoire; 4 Centre Suisse de Recherches Scientifiques en Côte d’Ivoire, 01 BP 1303, Abidjan 01, Côte d’Ivoire; 5 School of Biology, University of Nottingham, University Park, NG7 2RD, Nottingham, UK

**Keywords:** Schistosomiasis, *Schistosoma haematobium*, *Schistosoma mansoni*, Diagnosis, Antibodies, Cercariae, Sensitivity, Specificity, Positive predictive value, Negative predictive value, Côte d’Ivoire

## Abstract

**Background:**

Parasitological methods are widely used for the diagnosis of schistosomiasis. However, they are insensitive, particularly in areas of low endemicity, and labour-intensive. Immunoassays based on detection of anti-schistosome antibodies have the merit of high sensitivity and recently a rapid diagnostic test (RDT), incorporating *Schistosoma mansoni* cercarial transformation fluid (SmCTF) for detection of anti-schistosome antibodies in blood has been developed. Here, we assessed the diagnostic performance of the SmCTF-RDT for *S. mansoni* and *S. haematobium* infections by comparing it with microscopy for egg detection.

**Methods:**

A cross-sectional survey was carried out in Azaguié, south Côte d’Ivoire. 118 pre-school-aged children submitted two stool and two urine samples, which were subjected to the Kato-Katz and urine filtration methods for the detection of *S. mansoni* and *S. haematobium* eggs, respectively. Urine was also subjected to a commercially available cassette test for *S. mansoni*, which detects circulating cathodic antigen. A finger-prick blood sample was used for the SmCTF-RDT for detection of anti-*S. mansoni* and anti-*S. haematobium* antibodies.

**Results:**

The prevalence of both anti-*S. mansoni* and anti-*S. haematobium* antibodies was more than three times higher than the prevalence of infection estimated by egg detection under a microscope. Using quadruplicate Kato-Katz as the reference standard for the diagnosis of *S. mansoni* infection, the sensitivity, negative predictive value (NPV), and positive predictive value (PPV) of the SmCTF-RDT was 75.0%, 84.2% and 22.5%, respectively. When two urine filtrations were considered as the reference standard for the diagnosis of *S. haematobium* infection, the sensitivity, NPV and PPV of SmCTF-RDT was 66.7%, 94.9% and 5.1%, respectively. The specificity of SmCTF-RDT, when using egg-detection as the reference standard, was estimated to be 34.4%. This low specificity may be a reflection of the relative insensitivity of the direct diagnostic approaches using microscopy.

**Conclusions:**

The SmCTF-RDT is at least as sensitive as duplicate Kato-Katz and a single urine filtration for detection of *S. mansoni* and *S. haematobium*, respectively. Further investigations into the specificity of the test for anti-schistosome antibodies are necessary, but our results suggest that it may be a useful tool for mapping the prevalence of anti-schistosome antibodies in a given population pending intervention.

## Background

The diagnosis of schistosomiasis is traditionally achieved through the use of parasitological methods (urine filtration for *Schistosoma haematobium* and Kato-Katz thick smears for *S. mansoni* and *S. japonicum* infections). They are the most direct and specific way of detecting active infection, but often miss light-intensity infections [1-5], since only small amounts of excreta are examined. With preventive chemotherapy-based helminthiases control programmes due to be scaled up [6,7], helminth egg output is likely to decline further and the problem of insensitive egg counting methods will be exacerbated [8,9].

Considerable effort has been expended on the development of immunodiagnostic assays that will improve on microscopy. Circulating antigen detection assays are considered desirable since they are predicted most likely to reflect active infection. However, they have generally been shown to be no more sensitive than parasitological methods, particularly in situations where egg counts are low [10,11]. Validations of various formulations of a point-of-care (POC) assay to detect circulating cathodic antigen (CCA) in urine, including a commercially available POC-CCA cassette test in different African settings, have revealed that a single POC-CCA is at least as sensitive as a single Kato-Katz thick smear for the diagnosis of *S. mansoni*, but it is not useful for the diagnosis of *S. haematobium*[12-16].

With the lack of alternative tests available to detect infection directly, it is envisaged that antibody detection methods of diagnosis will become increasingly useful [2,4]. Indeed, they have been widely and successfully used in the national schistosomiasis control programme in the People’s Republic of China [17,18]. However, whilst they have the merit of high sensitivity [2], they are often criticised for their apparent lack of specificity and inability to distinguish active from past infection. ‘False antibody-positive’ results, particularly at the pre-treatment stage may, however, be a direct result of using insensitive parasitology as the reference standard.

A rapid diagnostic test (RDT) incorporating *S. mansoni* cercarial transformation fluid (SmCTF) (Vision Biotech; Cape Town, South Africa) has recently been developed following promising preliminary results on this antigen’s ability to detect anti-schistosome antibodies in an enzyme-linked immunosorbent assay (ELISA) format [19-21]. The antigen performed equivalently to schistosome soluble egg antigens in ELISA, an assay that is regularly employed in travellers’ medicine clinics for diagnosis [22,23] and has also been shown to perform well in schistosome-endemic areas [12,24]. This study was designed to determine the performance of the SmCTF-RDT for the diagnosis of * S. mansoni* and *S. haematobium* among preschool-aged children in a mixed infection focus of south Côte d’Ivoire in comparison with standard microscopy methods. This evaluation was integrated in a larger study pertaining to the diagnosis, epidemiology and treatment of schistosomiasis in preschool-aged children [25].

## Methods

### Ethical considerations

Ethical clearance was obtained from the Ministry of Health and Public Hygiene of Côte d’Ivoire (reference no. 4248/2010/MSHP/CNER). Village authorities were informed and the aims and procedures of the study were explained to the heads of households. We obtained written informed consent (or fingerprints for illiterates) from parents or legal guardians of the preschool-aged children [25]. At the end of the study, all preschool-aged children were given crushed praziquantel tablets (40 mg/kg, against schistosomiasis) and albendazole (200 mg, against soil-transmitted helminthiases), free-of-charge and irrespective of their infection status.

### Study area, design and sample size

The study reported here was part of the baseline cross-sectional survey carried out in August 2011 in the village of Azaguié M’Bromé (geographical coordinates: 05°39’42” N latitude and 04°08’38” W longitude), located some 50 km north of Abidjan, the economic capital of Côte d’Ivoire. *S. mansoni* and *S. haematobium* are co-endemic in the Azaguié district [25,26]. Villagers are mainly engaged in subsistence farming. Access to clean water and improved sanitation are lacking. Based on a previous investigation comparing the accuracy of two tests for the diagnosis of intestinal protozoa, we aimed at a minimum of 100 participants [27]. Allowing for a drop-out of 15–20%, we enrolled a total of 140 preschool-aged children (<6 years of age) who had not previously been treated for schistosomiasis.

### Inclusion criteria

We adhered to the following inclusion criteria: (i) children younger than 6 years of age; (ii) written informed consent by parents/guardians; (iii) submission of two sufficiently large stool samples for duplicate Kato-Katz thick smears per stool sample, and two urine samples for a 10 ml filtrate and a POC-CCA cassette test per urine sample; (iv) SmCTF-test result using a drop of whole blood from a finger-prick; (v) no recent schistosomiasis treatment (within the past 6 months) according to a parental questionnaire.

### Field and laboratory procedures

A door-to-door census was carried out in June 2011 to establish up-to-date census data. The data were used to generate lists of preschool-aged children, including their name, age, sex, and geographical coordinates of the household. Parents/guardians were invited to provide both a stool and urine sample from eligible children in the morning (between 08:00 and 12:00 hours) on each of two consecutive days. Urine, stool and blood samples were collected at a central locality in the village. Additionally, children’s height and weight were measured. Testing of SmCTF-RDTs took place on-site. The urine and stool samples were transferred to a nearby laboratory and diagnostic work-up completed the same day.

From each stool sample, duplicate Kato-Katz thick smears (using standard 41.7 mg templates) were prepared [28]. After a clearing time of at least 30 min, the slides were examined under a microscope by one of four experienced technicians and the number of *S. mansoni* (and other helminth) eggs counted. Ten millilitres of each urine sample was filtered (pore aperture 20 μm; Sefar AG, Heiden, Switzerland), placed on a slide and examined under a microscope for *S. haematobium* eggs. Additionally, a drop of urine was placed on a POC-CCA cassette (batch no.: 33112: Rapid Medical Diagnostics; Pretoria, South Africa) and the results recorded as either negative, trace or positive. If a trace was reported on only one out of the two days, this was taken to be a negative result, otherwise trace results were taken to be positive [15].

On the first day of sampling, one drop of finger-prick blood was taken and added to the sample well of the SmCTF-RDT cassette, followed by a few drops of buffer. Test results were read after 15 min; colour development of the control band indicated the test was not faulty, and a test band indicated if the result was positive. SmCTF was prepared by BioGlab Ltd., (Nottingham, UK) using a previously described method [21]. It was freeze-dried and forwarded to Vision Biotech (Cape Town, South Africa) for incorporation into RDT-format cassettes.

### Statistical analysis

**Figure 1:**
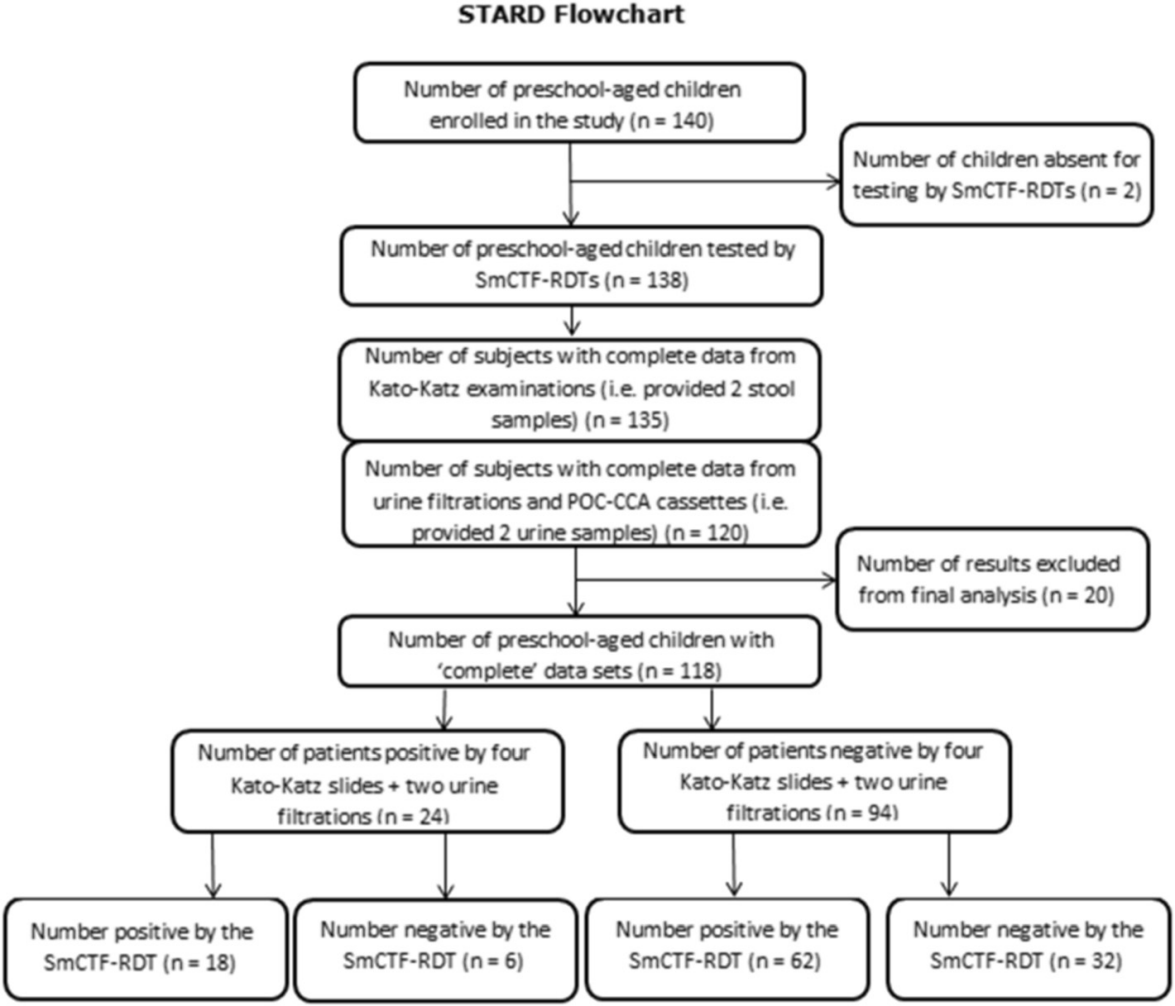
**STARD flowchart detailing the study participation and adherence of preschool-aged children for submission of stool and urine samples.** The study was carried out in Azaguié M’Bromé, south Côte d’Ivoire between June and September 2011. From those preschool-aged children who provided complete data, the results obtained by the SmCTF-RDT were compared to parasitological methods for diagnosis of *S. mansoni* and *S. haematobium* infections.

Data were double-entered into an Excel spreadsheet, transferred into EpiInfo version 3.2 (Centers for Disease Control and Prevention; Atlanta, USA), and cross-checked. Only children who had complete data from the baseline surveys (i.e. quadruplicate Kato-Katz thick smears, two POC-CCA cassette tests, two urine filtrations and a single SmCTF cassette test) were included in the final analysis (n = 118; Figure 1). Figures were carried out using GraphPad Prism version 5 (GraphPad Software, Inc., La Jolla, USA) and statistical analysis were performed using SPSS version 16 (IBM Corp., New York, USA).

Dichotomous data (e.g. presence or absence of an infection) are presented as a proportion. For determining the SmCTF cassette test accuracy, two reference standards were used: (i) results of quadruplicate Kato-Katz thick smears for *S. mansoni*, and (ii) results of two urine filtrations for *S. haematobium*. Thus, the SmCTF cassette test was compared with Kato-Katz for the diagnosis of *S. mansoni* and with a urine filtration method of egg detection for *S. haematobium* diagnosis. Sensitivity was calculated as the number of true-positives (TP) / (TP + false-negatives (FN)), specificity as true-negatives (TN) / (TN + false-positives (FP)), positive predictive value (PPV) as TP / (TP + FP), and negative predictive value (NPV) as TN / (TN + FN) [29].

The relationship between the binary SmCTF-RDT result and socio-demographic characteristics (e.g. age, sex, height and weight of children) was assessed using univariate logistic regression analysis. Test results were also compared to schistosome egg count per gram of faeces (EPG) and the egg counts of other helminths (*Ascaris lumbricoides*, *Trichuris trichiura* and hookworm) using logistic regression analysis.

## Results

### Study adherence and population characteristics

The STARD flowchart (Figure 1) shows that a total of 140 preschool-aged children were enrolled in Azaguié M’Bromé. Complete diagnostic data (i.e. quadruplicate Kato-Katz thick smears, duplicate POC-CCA cassette tests, duplicate urine filtrations and a single SmCTF-RDT) were available for 118 children. There were 62 girls (52.5%) and 56 boys with a mean age of 3.4 years (range: 3 months to 5.5 years) (Table 1). No association was observed between SmCTF-RDT results and the socio-demographic factors listed in Table 1 (all p >0.05).

**Table 1:** Baseline characteristics of the study participants (n = 118)

**Characteristics**	**n (%)**	**χ**^**2**^	**p-value**
**Sex**			
Male	56 (47.5)		
Female	62 (52.5)	0.200	0.652
**Mean age (range)**	3.4 years (3 months – 5.5 years)		
Mean haemoglobin, g/dl (range)	10.3 (6.5–16.0)		
Mean height, cm (range)	92 (51, 122)		
Mean weight, kg (range)	13.2 (3.3, 20.6)		

### Prevalence and intensity of schistosome infections

Of the 118 preschool-aged children with complete data records, 24 were positive for *S. mansoni* infections by quadruplicate Kato-Katz thick smear examinations (two stool samples, each subjected to duplicate Kato-Katz), giving an infection prevalence of 20.3%. Of the 24 *S. mansoni* egg-positive children, 70.8% (n = 17) were lightly infected (1–99 EPG), 20.8% (n = 5) had moderate infection intensity (100–399 EPG) and 8.4% (n = 2) had heavy infections (≥ 400 EPG). The mean faecal egg count of infected children was 98.7 EPG (95% confidence interval (CI) 32.3–165.0 EPG). There was no association between faecal egg counts and binary SmCTF-RDT results (p >0.05).

The prevalence of *S. mansoni* infections estimated by a single POC-CCA was nearly three times higher than that estimated by quadruplicate Kato-Katz at 51.7%. Prevalence estimated by two POC-CCA was 63.6%.

Only six preschool-aged children were deemed to be infected with *S. haematobium* by two urine filtrations, hence the prevalence was 5.1%. There were, on average, <2 eggs seen per 10 ml of urine in the egg-positive children. Five of these six *S. haematobium* cases were concurrently infected with *S. mansoni*.

Prevalence of both *S. mansoni* and *S. haematobium* infections estimated by quadruplicate Kato-Katz and two urine filtrations was 21.2% (95% CI 14.4–29.9%).

The prevalence of anti-schistosome antibodies as estimated by the SmCTF-RDT was more than three times higher (66.9%; 95% CI 57.6–75.2%).

### Prevalence of other helminths

The observed prevalence of *T. trichiura*, hookworm and *A. lumbricoides* in the population was 14.4%, 10.2% and 5.1%, respectively. Of the schistosome egg-negative individuals, there were 10 positive for *T. trichiura*, six for hookworm and five for *A. lumbricoides*. Six, four and three of these were positive by the SmCTF-RDT, respectively. There was no association found between SmCTF-RDT results and the presence/absence of other helminth infections (all p >0.05).

### Diagnostic performance of the SmCTF-RDT

Using results from the quadruplicate Kato-Katz thick smears as the reference standard for comparison, the sensitivity of the SmCTF-RDT for *S. mansoni* diagnosis was 75.0% (95% CI: 52.9–89.4%). This result was comparable to that achieved by duplicate Kato-Katz thick smears (prepared from first day stool sample), and a duplicate POC-CCA cassette (Table 2). Sensitivity of a single Kato-Katz slide was only 50.0% (95% CI 29.6–70.4%) when compared with four Kato-Katz slides.

**Table 2:** **Sensitivity, specificity, negative predictive value (NPV) and positive predictive value (PPV) of each diagnostic assay using quadruplicate Kato-Katz thick smears as the reference standard for *S. mansoni *diagnosis and two urine filtrations as the reference standard for *S. haematobium *diagnosis**

**Four Kato-Katz as reference standard**	**Sensitivity (95% CI)**	**Specificity (95% CI)**	**NPV (95% CI)**	**PPV (95% CI)**
Single Kato-Katz thick smear (day 1)	50.0 (29.6–70.4)	100 (95.1–100)	88.7 (80.7–93.8)	100 (69.9–100)
Duplicate Kato-Katz thick smears	75.0 (52.9–89.4)	100 (95.1–100)	94.0 (86.9–97.5)	100 (69.2–100)
Single POC-CCA cassette (day 1)	75.0 (52.9–89.4)	53.2 (42.7–63.5)	89.3 (77.4–95.6)	29.0 (18.6–42.1)
Duplicate POC-CCA cassettes	75.0 (52.9–89.4)	46.8 (36.5–57.3)	88.0 (75.0–95.0)	26.4 (16.8–38.8)
Single SmCTF-RDT	75.0 (52.9–89.4)	34.0 (24.8–44.6)	84.2 (68.1–93.4)	22.5 (14.2–33.5)
**Two urine filtrations as reference standard**	**Sensitivity (95% CI)**	**Specificity (95% CI)**	**NPV (95% CI)**	**PPV (95% CI)**
Single urine filtration (day 1)	66.7 (24.1–94.0)	100 (95.9–100)	98.2 (93.2–99.7)	100 (39.6–100)
Single SmCTF-RDT	66.7 (24.1–94.0)	33.0 (24.6–42.6)	94.9 (81.4–99.1)	5.1 (1.6–13.1)

The study was carried out in Azaguié M’Bromé, south Côte d’Ivoire between June and September 2011 and focussed on a subset of 118 preschool-aged children (<6 years of age).

CI, confidence interval.

Of the six *S. haematobium*-positive children, four were also positive by the SmCTF-RDT, giving a sensitivity of 66.7% (95% CI 24.1–94.0%). This result was comparable to that achieved by a single urine filtration (sensitivity = 66.7%, 95% CI 24.1–94.0%). One of the *S. haematobium* infections missed by the SmCTF-RDT was a subject co-infected with *S. mansoni*.

Specificity of the SmCTF-RDT for the diagnosis of *S. mansoni* was calculated at 34.0% (95% CI 24.8–44.6%) when using four Kato-Katz thick smears as the reference standard (Table 2). For *S. haematobium* infections, specificity of SmCTF-RDT, compared to two urine filtrations, was 33.0% (95% CI 24.6–42.6%). Specificity to either schistosome infection, when using the quadruplicate Kato-Katz thick smears and two urine filtrations as the reference standards, was 34.4% (95% CI 25.1–45.1%).

The NPV and PPV of a single SmCTF-RDT, compared to quadruplicate Kato-Katz, was 84.2% and 22.5%, respectively. The NPV and PPV of a single SmCTF-RDT, compared to two urine filtrations, was 94.9% and 5.1%, respectively.

**Figure 2:**
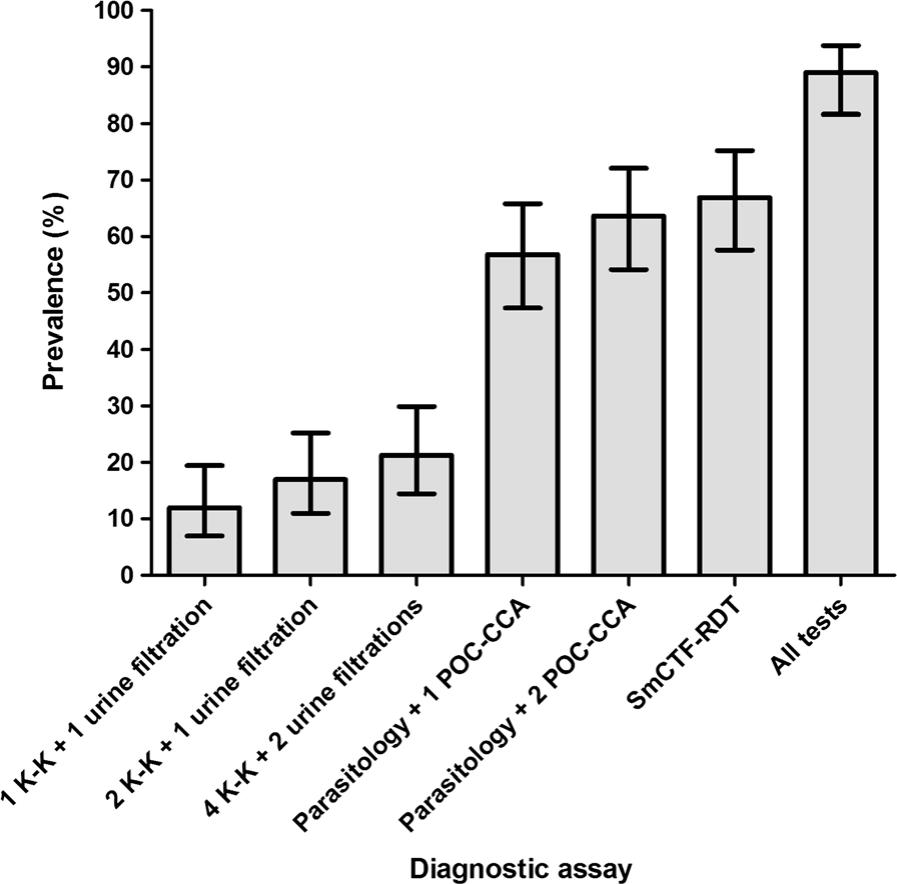
**Prevalence of schistosome infections in preschool-aged children according to different diagnostic methods and sampling efforts.** The study was carried out in Azaguié M’Bromé, south Côte d’Ivoire in August 2011. Prevalence was estimated by each indicated diagnostic assay (K-K, Kato-Katz; POC-CCA, point-of-care circulating cathodic antigen; SmCTF-RDT, *S. mansoni* cercarial transformation fluid rapid diagnostic test).

The prevalence of schistosome infections estimated by each diagnostic assay, or combinations thereof, is summarised in Figure 2. With increasing numbers of parasitological examinations, the estimated prevalence increased, but still only to a much lower level than that estimated by both the POC-CCA cassette and the SmCTF-RDT. When all tests were combined, the estimated prevalence increased to 89.0% (95% CI 81.6–93.8%), indicating that agreement between the ‘false-positive’ results achieved with the POC-CCA cassette and those with the SmCTF-RDT was relatively low. Many of the SmCTF-RDT positive, POC-CCA negative results may be attributed to the SmCTF-RDT’s ability to detect anti-*S. haematobium* antibodies.

## Discussion

This study was designed to evaluate the diagnostic potential of a RDT (SmCTF-RDT) which is capable of detecting antibodies with specificity for the two main *Schistosoma* species occurring in Africa, *S. mansoni* and *S. haematobium*. Here we have determined – for the first time – the performance of the SmCTF-RDT among preschool-aged children who are known to have mainly low intensity schistosome infections [30-32], which may be analagous to populations subjected to repeated rounds of preventive chemotherapy such as in areas targeted for schistosomiasis elimination [33].

We found that of the 118 preschool-aged children with complete data records, prevalence of *S. mansoni* and *S. haematobium* infections, according to egg-detection methods, was 20.3% and 5.1%, respectively. Around 70% of the children infected harboured light infections. This is in agreement with recent studies that have shown low prevalence and mainly light intensity infections in preschool-aged children [30-32]. However, egg-detection methods are known to lack sensitivity, particularly when infection intensities are light [1-5,34]. The SmCTF-RDT employed here estimated that the prevalence of *S. mansoni* and *S. haematobium* infections was 66.9%, more than three times higher than that estimated by egg-detection methods. Single and duplicate POC-CCA tests estimated prevalence of *S. mansoni* infections to be 51.7% and 63.6%, respectively.

This study showed that a single SmCTF-RDT is more sensitive than a single Kato-Katz thick smear and at least as sensitive as duplicate Kato-Katz thick smears for the diagnosis of *S. mansoni* infection. It is also as sensitive as one urine filtration for the diagnosis of *S. haematobium* infection.

Specificity of the SmCTF-RDT was low compared to egg-detection methods, but this may be a direct consequence of their relative insensitivity. The specificity of the POC-CCA test was also low when using microscopy as the reference standard. There was little agreement between the ‘false-positive’ results seen with each assay and so this raises the question: which of the ‘false-positive’ results, by either the SmCTF-RDT or the POC-CCA, are in fact ‘true-positives’? Comparison of these two immunoassays with a potentially more accurate reference test (e.g. polymerase chain reaction, PCR) may provide an answer [35,36].

Whilst the traditional parasitological methods employed here have their advantages, such as high specificity, provision of a quantitative measure of infection, and the Kato-Katz has the ability to concurrently diagnose *S. mansoni* and soil-transmitted helminths [4,5,37], they are insensitive, laborious and relatively expensive to perform [38]. For large-scale schistosomiasis control programmes, a RDT is much more likely to be useful, particularly one that meets the ASSURED criteria for diagnostic tests [39]. Both the SmCTF-RDT and POC-CCA are user-friendly, rapid, equipment-free and deliverable. They can be read by non-specialized individuals with limited training, they do not require use of a laboratory or even electricity, and they also give results within 20 min allowing diagnosis at the point-of-care.

An advantage of the commercially-available POC-CCA test over the SmCTF-RDT is that it works with urine rather than blood and so is less invasive. Since it detects circulating antigen rather than antibody, it is also able to discriminate between active and past infections. However, whilst the POC-CCA test is estimated to be at least as sensitive as one Kato-Katz thick smear for the diagnosis of *S. mansoni* infection, it unfortunately does not work well for the diagnosis of *S. haematobium* infections. The SmCTF-RDT, whilst unable to distinguish between them, has the ability to detect both *S. mansoni* and *S. haematobium* infections. Many areas of sub-Saharan Africa are co-endemic for both schistosome species, and so a test able to detect both infections will be particularly useful. Another downfall of the POC-CCA cassette is its cost, currently around US$ 1.75 per test. The SmCTF-RDT employed here is not yet commercially available, but it is estimated that it will cost less than US$ 1 per test.

## Conclusions

The SmCTF-RDT meets the ASSURED criteria for diagnostic tests [39] and could be a useful tool for estimating the prevalence of schistosome infections prior to intervention. Here, the test performed well for the diagnosis of both *S. mansoni* and *S. haematobium* infections in a preschool-aged population. Sensitivity of the SmCTF-RDT for *S. mansoni* was comparable to both duplicate Kato-Katz slides and a duplicate POC-CCA cassette. Sensitivity for *S. haematobium* was comparable to a single urine filtration, but only six preschool-aged children were infected with this species and five of these were also concurrently infected with *S. mansoni*. Future evaluations should, therefore, use larger sample sizes and also investigate performance across different settings, e.g. a setting endemic for *S. mansoni* infection only, another for *S. haematobium* infection only and a third, mixed infection focus. Specificity was relatively poor when using egg-detection methods as the reference standard. It is predicted that when using a standard that is more sensitive, specificity will be much higher than reported here. For future evaluations to be more accurate, the number of egg counts should be increased further, or alternatively, potentially more accurate techniques such as PCR be employed [35,36]. Evaluations in areas non-endemic for schistosomiasis will also be necessary to assess whether there is cross-reactivity with antibodies against other helminth infections.

## Competing interests

MJD is the owner of BioGlab Ltd. that produced the SmCTF employed here in the RDT format. All other authors have no competing interests.

## Authors’ contributions

JTC, EKN and JU conceived and designed the experiments; JTC and EKN performed the experiments; MJD provided the SmCTF used in this study; JTC and EMD analysed the data; JTC, JU, MJD and EMD prepared the manuscript. All authors have read and approved the final version of the manuscript.
